# Analysis and Identification of Therapeutic Targets for Neuronal Regeneration After Ischemic Stroke

**DOI:** 10.1002/brb3.70377

**Published:** 2025-05-05

**Authors:** Xiao‐Li Min, Li Guo, Zhenyu Wang, Lei Zhao, Chenglong Shi, Xiaoyong Liu, Zhen Wang, Fei‐Fei Shang, Jiaping Wang

**Affiliations:** ^1^ Department of Cerebrovascular Diseases The Second Affiliated Hospital of Kunming Medical University Kunming China; ^2^ Department of Radiology The Second Affiliated Hospital of Kunming Medical University Kunming China; ^3^ Department of Cardiology The Second Affiliated Hospital of Kunming Medical University Kunming China; ^4^ Department of Neurosurgery The Second Affiliated Hospital of Kunming Medical University Kunming China; ^5^ Institute of Life Science Chongqing Medical University Chongqing China

**Keywords:** ischemic stroke, neuronal regeneration, target genes

## Abstract

**Objective:**

The aim of this study is to analyze and identify genes associated with neuronal regeneration after ischemic stroke (IS) and to predict potential therapeutic targets for neuronal regeneration after IS using bioinformatics analysis methods.

**Methods:**

The GSE137482 and GSE208121 datasets were obtained from the Gene Expression Omnibus (GEO) database, and the differentially expressed hub genes that showed decreased expression in GEO and increased expression in neuronal regeneration after IS were identified as key genes. To identify the key genes, functional enrichment and Protein–Protein Interaction (PPI) network analysis were conducted. The expression levels of the key genes were characterized by real‐time quantitative polymerase chain reaction (RT‐qPCR) and western blot in neuron‐induced cell models. Additionally, possible regulatory networks of the key genes were analyzed.

**Results:**

The screening process yielded 24 differentially expressed pivotal genes, which were predominantly enriched in processes related to epithelial cell proliferation regulation and hormone response. The PPI analysis yielded five key genes (Npas4, Nr4a3, Nr4a1, Egr4, and Egr1), which may exert regulatory roles primarily through peptide and peptide hormone responses. RT‐qPCR and western blot assays confirmed that the expression levels of the key genes were elevated in the neuron‐like differentiated cell model. However, these findings were inhibited by additional treatment with hypoxia. The analysis of the key gene regulatory network revealed that EGR1 and NR4A1 might regulate hub genes by utilizing their transcription factor properties, with EGR1 being the predominant regulator. The validation results from our cellular model indicated that upregulating EGR1 promotes neuronal‐like differentiation in SH‐SY5Y cells.

**Conclusion:**

EGR1 could potentially serve as a therapeutic target for neuronal regeneration following IS.

## Graphical abstract

In this study, we screened and analyzed targets associated with neuronal regeneration after ischemic stroke (IS), and detected the expression of corresponding genes in cellular models. Our findings suggest that EGR1 and NR4A1 may represent promising therapeutic targets of neuronal regeneration after IS.

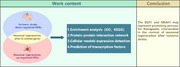

## 1 Introduction

Ischemic stroke (IS) is a focal neurologic deficit disease that is caused by arterial occlusion due to thrombosis or embolism. It accounts for 60%–70% of all stroke types (Hilkens et al. 2024). IS is characterized by acute and persistent cerebral ischemia and hypoxia, IS results in a detrimental disruption of brain blood circulation, leading to severe and often irreversible consequences (Hankey 2017). It has been reported that IS is directly leading to 5.9 million deaths and indirectly resulting in 102 million disability‐adjusted deaths each year (Qin et al. 2022). The following factors have been identified as potential risk factors for IS: diabetes mellitus, smoking, hyperlipidemia, and hypertension (Kleindorfer et al. 2021). The main known causes include atherosclerotic plaques and atherosclerotic plaque ruptures of the cerebral vessels, cardiac cerebral infarcts, and lacunar infarcts of small vessel disease (Bailey et al. 2012; Feigin et al. 2014). Timely clinical intervention to restore cerebral perfusion is the main strategy for treating IS. This involves promoting revascularization through intravenous thrombolysis, mechanical thrombectomy in arteries, or a combination of both approaches (Hankey 2017; Anfray et al. 2021). However, due to limitations such as short time windows and risks of bleeding (Hu et al. 2024), the efficacy of these treatments has not been satisfactory, which is an important cause of death or disability among patients. Furthermore, antithrombotic treatment with anticoagulants or antiplatelet agents is required following IS to reduce the risk of potential recurrence (Kleindorfer et al. 2021), imposing a long‐term life and economic burden on patients.

IS can easily lead to widespread neuronal death, brain tissue infarction, and subsequent neurological deficits, increasing the risk of cognitive impairments (Powers 2020; Levine et al. 2015). Therefore, the recovery of neurological function has gradually become one of the hotspots in the treatment of IS. The main strategies to promote neurofunctional recovery include repairing and regenerating damaged neurons (Barker et al. 2018), especially the regeneration of neurons has attracted considerable attention. Previous studies have reported promising methods for neuronal regeneration. For example, Yin's developed a mixture composed of 3–4 small molecules, which successfully induced human fetal astrocytes into functional neurons through this compound (Yin et al. 2019). Ninomiyas team used a mixture of six small molecules to convert macrophages into neuronal cells (Ninomiya et al. 2023). Neural stem cell therapy replaces damaged neurons through homing and cellular replacement effects, secretes extracellular vesicles to protect and induce neuronal regeneration, and converts astrocytes into neurons, among other mechanisms (Wang et al. 2024). These methods of neuronal regeneration have brought new hope, but complex microenvironments after IS, survival rates of regenerated neurons, and other issues still exist. Identifying potential therapeutic targets for neuronal regeneration may help to explore more effective techniques for inducing neuronal regeneration. Therefore, this study aims to analyze and discuss potential therapeutic targets for neuronal regeneration after IS to provide a new perspective for the treatment of neuronal regeneration after IS.

## 2 Materials and Methods

### 2.1 Analysis of Differentially Expressed Genes

The GEO database (https://www.ncbi.nlm.nih.gov/gds, RRID: SCR005012) was accessed to obtain the GSE137482 and GSE208121 datasets. The GSE137482 dataset used 3‐ and 18‐month‐old C57Black/6 mice to simulate brain ischemia via permanent middle cerebral artery occlusion (MCAO). Tissue from the parietal cortex was collected 3 days post MACO for RNA‐seq analysis. According to the description in the original study, the transcriptional response to stroke in the 3‐ and 18‐month‐old brain is highly similar and differs primarily in magnitude. To exclude interference caused by aging, we opted to use the sequencing data from the 3‐month‐old group as the IS group for differential analysis. The GSE208121 dataset utilized erinacines (ES) derived from *Hericium erinaceum* to induce neuronal regeneration in primary cortical neurons of mice, followed by RNA‐seq analysis of cells at 16 and 48 h post‐induction. According to the original results, we selected the sequencing data of the 48‐h group, where neuronal regeneration was most pronounced, as the data for the neuronal regeneration group. Using the GEOquery V2.64.2 package (RRID: SCR000146) in R V4.3.3 software (RRID: SCR001905), we obtained the required data and performed differential analysis using the limma V3.52.2 package (RRID: SCR010943). We set the threshold for differentially expressed genes (DEGs) as *p* < 0.05 and |logFC| > 0.58. The TBtoolsII V2.119 (Chen et al. 2023) (RRID: SCR023018) was used to create a Venn diagram to screen for MACO downregulated and ES upregulated intersecting genes. The expression profiles of hub genes were visualized using the ggplot2 V3.3.6 package (RRID: SCR014601).

### 2.2 Functional Enrichment Analysis

The clusterProfiler V4.0 package (RRID: SCR016884), org.Mm.eg.db package (RRID: SCR023488), and GOplot V1.0.1 package (RRID: SCR024419) were used to perform Gene Ontology (GO) and Kyoto Encyclopedia of Genes and Genomes (KEGG) analyses on the hub genes involved in neuronal regeneration after IS.

### 2.3 Protein–Protein Interaction Network Analysis

Upload the list of hub genes to the STRING database (https://cn.stringdb.org/, RRID: SCR005223) for protein–protein interaction network PPI analysis. The analysis results were imported into Cytoscape V3.10.0 software (RRID: SCR003032), and the MCODE plugin (RRID: SCR015828) was used for clustering analysis (degree cutoff = 2, node score cutoff = 0.2, KCore = 2, and max. depth = 100) to obtain key clusters. The CytoNCA plugin was used for parameter analysis of the key clusters.

### 2.4 Neuronal Induction of SHSY5Y Cells

The SHSY5Y cell line was purchased from Wuhan Puyuesi Biotechnology Co., Ltd. (Cat#CL0208, RRID: CVCL0019). The cells were cultured in DMEM medium (Gibco, Cat#11966025) supplemented with glutamine (2 mM/L), penicillin (20 units/mL), streptomycin (20 mg/mL), and 15% heat inactivated fetal bovine serum FBS (Gibco, Cat# 26010066). All cells were cultured in a humidified incubator containing 95% air and 5% CO_2_ at 37°C. The hypoxia treated group was cultured in a humidified incubator containing 5% CO_2_, 1% O_2_, and 94% N_2_ at 37°C. Neuronal‐like induction was performed according to the method described by Encinas (Encinas et al. 2000). Specifically, cells were seeded at an initial density of 10^4^ cells/cm^2^ in culture dishes previously coated with 0.05 mg/mL collagen. All‐trans retinoic acid (ATRA) 10 µM (Sigma–Aldrich, Cat#R2625) was added to the culture medium. After cell adhesion, the culture medium was switched to that containing ATRA to induce neuronal‐like differentiation for 5 days. The cells were washed three times with DMEM, and neuronal‐like differentiation was observed under a microscope.

### 2.5 Quantitative Real‐Time Polymerase Chain Reaction (qRT‐PCR)

Total RNA was extracted from SH‐SY5Y cells using the RNAiso Plus kit (Takara, Cat#9108). Following treatment with Recombinant DNase I (Takara, Cat#2270A), the RNA samples were reverse transcribed according to the instructions provided in the One Step PrimeScript RT‐PCR Kit (Takara, Cat#RR064A). The reaction system was prepared, and qRT‐PCR was performed on an ABI 7500. The primers used are listed below.

NPAS4‐F: 5‐GCCCTGCCTCGCTCTTCC‐3,

NPAS4‐R: 5‐TCTCTGCCTGAATATCTCCACTCTC‐3;

NR4A1‐F: 5‐TTCATGGACGGCTACACAGGAG‐3,

NR4A1‐R: 5‐GGTGGCTGAGGACGAGGATG‐3;

NR4A3‐F: 5‐GCAACTACGAACTCAAGCCTTCC‐3,

NR4A3‐R: 5‐TGGTGGTGGTGGTGGTGATG‐3;

EGR1‐F: 5‐TGGAGGAGATGATGCTGCTGAG‐3,

EGR1‐R: 5‐GCTGCTGCTGCTGCTGTTG‐3;

EGR4‐F: 5‐CCCAGCCAACAGACTCTATCCC‐3,

EGR4‐R: 5‐CGCCGTCGCCGCTACTC‐3;

GAPDH‐F: 5‐CTGGGCTACACTGAGCACC‐3,

GAPDH‐R: 5‐AAGTGGTCGTTGAGGGCAATG‐3.

### 2.6 Western Blot

Centrifuge the harvested cells and add RIPA lysis buffer (Thermo Scientific, Cat# 89901) to lyse the cells. Determine protein concentrations using the BCA Protein Assay Kit (Thermo Scientific, Cat#23227). Separate 20 µg sample proteins using 10% SDS‐PAGE and transfer the proteins to PVDF membranes. Seal the membranes with 5% skim milk for 1 h, wash with Tris‐buffered saline, and incubate with primary antibodies overnight at 4°C. After washing with Tris‐buffered saline, incubate with horseradish peroxidase (HRP)‐conjugated secondary antibodies at room temperature for 1 h. Use SuperSignal West Pico PLUS (Thermo Scientific, Cat# 34577) detection strips to visualize the bands. Analyze the band gray values using ImageJ (RRID: SCR_003070), and normalize the expression levels using GAPDH expression levels. The information about the antibodies used is as follows: anti‐NPSA4 (abcam, Cat#ab109984), anti‐NR4A1 (abcam, Cat#ab153914), anti‐NR4A3 (abcam, Cat# ab313781), anti‐EGR1 (abcam, Cat# ab196301), anti‐EGR4 (abcam, Cat# ab198197), goat anti‐rabbit IgG H&L (HRP) (abcam, Cat#ab97051), and donkey anti‐goat IgG H&L (HRP) (abcam, Cat#ab97110).

### 2.7 Prediction of Transcription Factors

Obtain the human transcription factor list through the AnimalTFDB v4.0 database (https://guolab.wchscu.edu.cn/AnimalTFDB4/#/, RRID: SCR_001624). Use TBtools‐II to create a Venn diagram to screen for transcription factors in the key clusters. Predict the target genes of the screened transcription factors using the TFLink database (https://tflink.net/), and use TBtools‐II to create a Venn diagram to screen for the core genes regulated by the transcription factors. Visualize the regulatory relationships between transcription factors and core genes using Cytoscape V3.10.0.

### 2.8 Transfection and Culture of SH‐SY5Y Cells

SH‐SY5Y cells were seeded in six‐well plates and allowed to adhere. Following cell attachment, transfection was performed using Lipofectamine 3000 with EGR1 overexpression plasmid (OE‐EGR1) and empty plasmid (OE‐NC). Plasmid dilution: 1 µg of plasmid was diluted in 5 µL of serum‐free culture medium, gently mixed, and incubated at room temperature for 5 min. Lipofectamine 3000 dilution: 3 µL of Lipofectamine 3000 was diluted in 5 µL of serum‐free culture medium, gently mixed, and incubated at room temperature for 5 min. The plasmid and Lipofectamine 3000 were then gently mixed, incubated at room temperature for 20 min, and added to the six‐well plates. The cells were then placed in the incubator for 48 h.

After confirming the EGR1 expression levels through Western blot detection, the cells were routinely cultured for an additional 5 days. The neuronal‐like differentiation of the cells was observed under the microscope.

### 2.9 Statistical Analysis

Data are presented as SEM ± mean value and analyzed using GraphPad Prism 9 (RRID: SCR_002798). A one‐way ANOVA was used for statistical differences, and Student's *t*‐test was used to analyze differences between the two groups. A *p* value < 0.05 was considered statistically significant.

## 3 Results

### 3.1 Screening of Genes Associated With Neuronal Regeneration After IS

**FIGURE 1:**
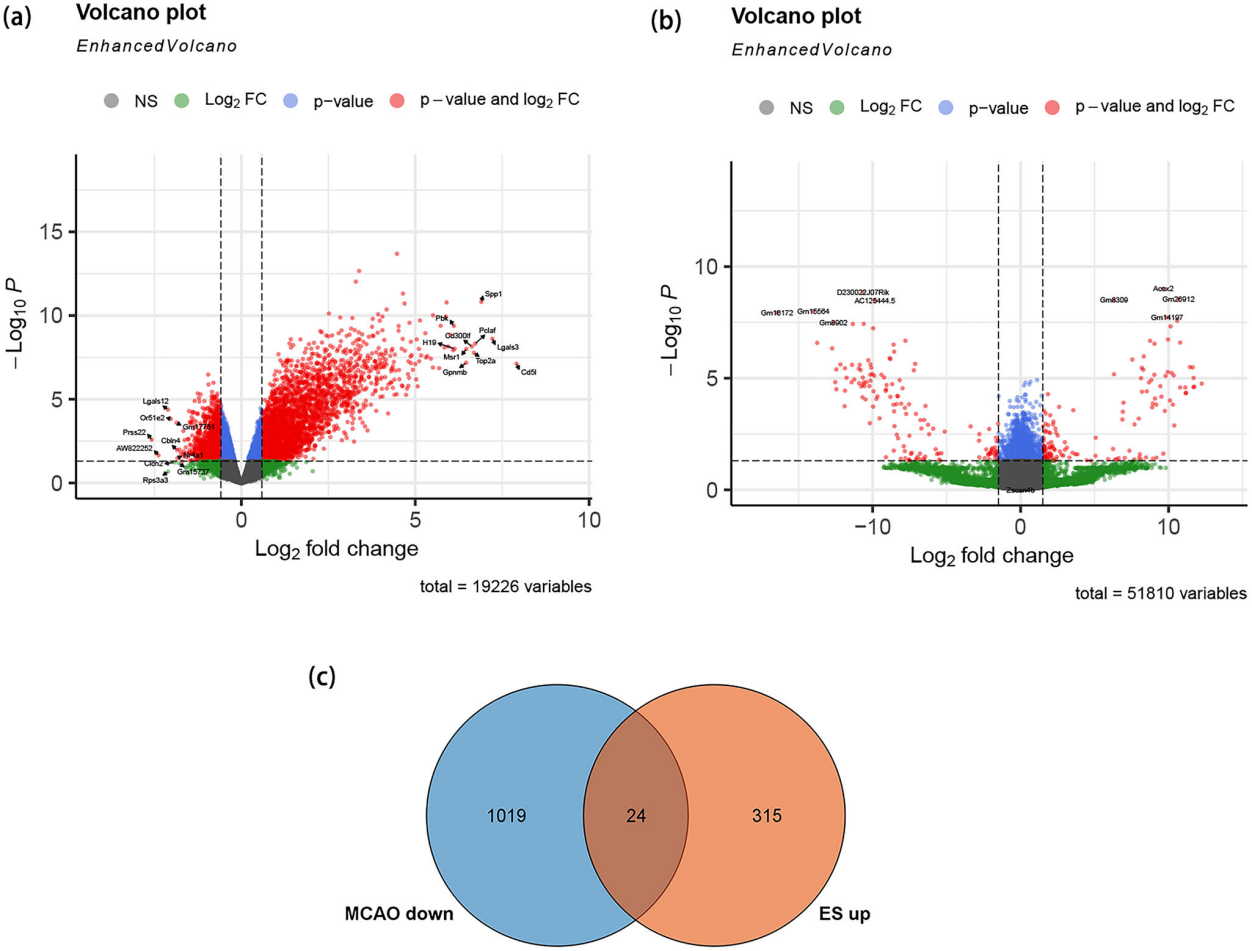
Screening of neuronal regeneration‐related genes after IS. (a) Volcano plot of DEGs in the GSE137482 dataset. (b) Volcano plot of DEGs in the GSE208121 dataset. (c) Venn diagram of downregulated and upregulated DEGs after MCAO and ES treatment. DEGs, differentially expressed genes; ES, erinacines; IS, ischemic stroke; MCAO, middle cerebral artery occlusion.

**FIGURE 2:**
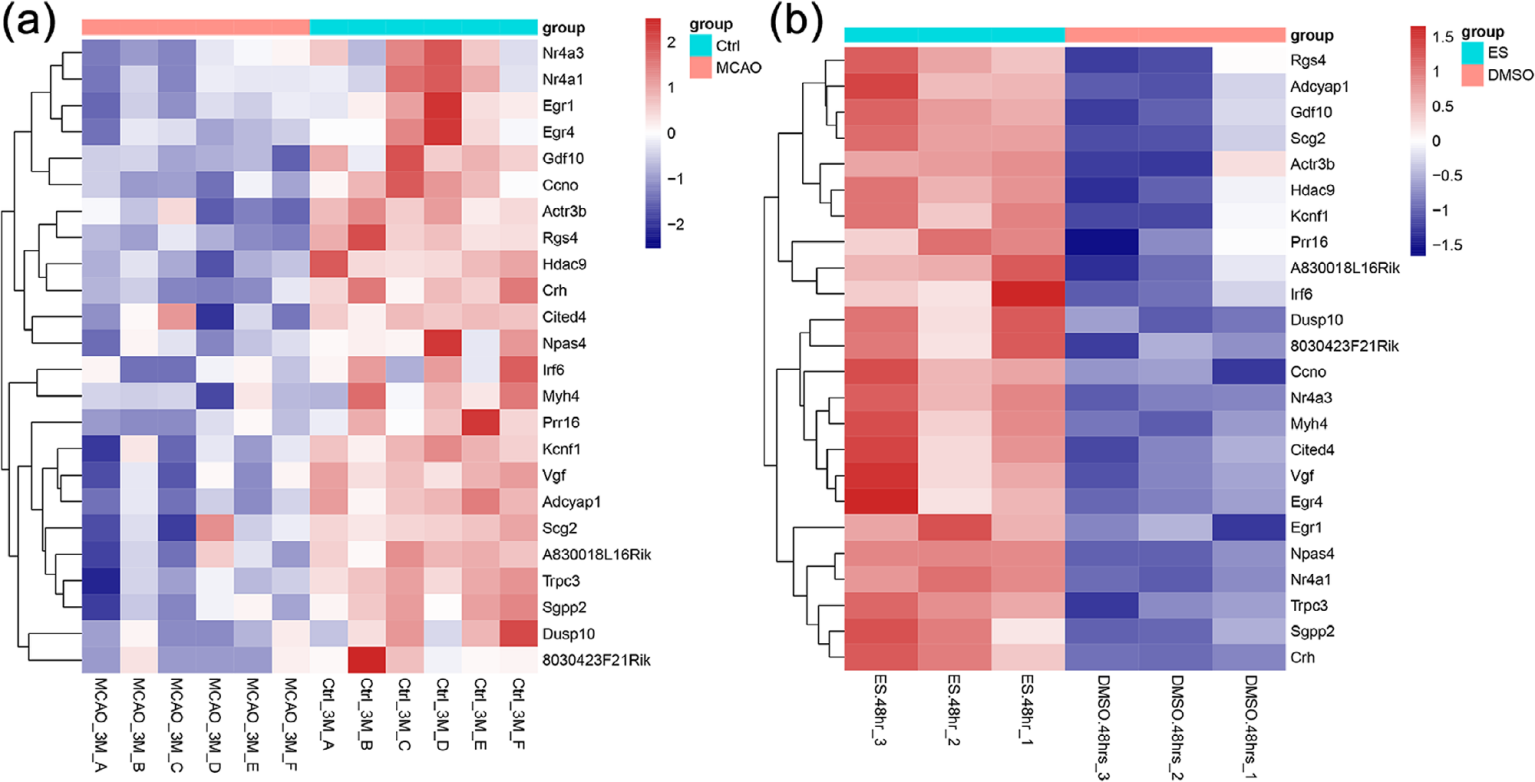
Heatmaps of gene expression profiles related to neuronal regeneration after ischemic stroke (IS). (a) Heatmap showing the expression of hub genes in the GSE137482 dataset. (b) Heatmap showing the expression of hub genes in the GSE208121 dataset.

In accordance with the screening criteria delineated in the experimental methodology, the two GEO datasets (GSE137482, GSE208121) were subjected to screening for DEGs. A total of 3976 DEGs were identified in the dataset following IS genesis, of which 1043 were found to be downregulated (Figure 1a). The GSE208121 dataset identified 618 DEGs involved in neuronal regeneration correlation, of which 339 DEGs were upregulated (Figure 1b). To elucidate the genes that facilitate neuronal regeneration and were suppressed by the occurrence of IS, the intersection of IS downregulation and neuronal regeneration upregulation was identified through the use of Venn plots. We obtained 24 genes related to neuronal regeneration after IS, and these 24 genes were defined as hub_genes (Figure 1c). Subsequently, the expression levels of the hub_gene in both datasets were depicted through the use of heatmaps (Figure 2a,b).

### 3.2 GO Enrichment of Neuronal Regeneration After IS‐Related DEGs

**FIGURE 3:**
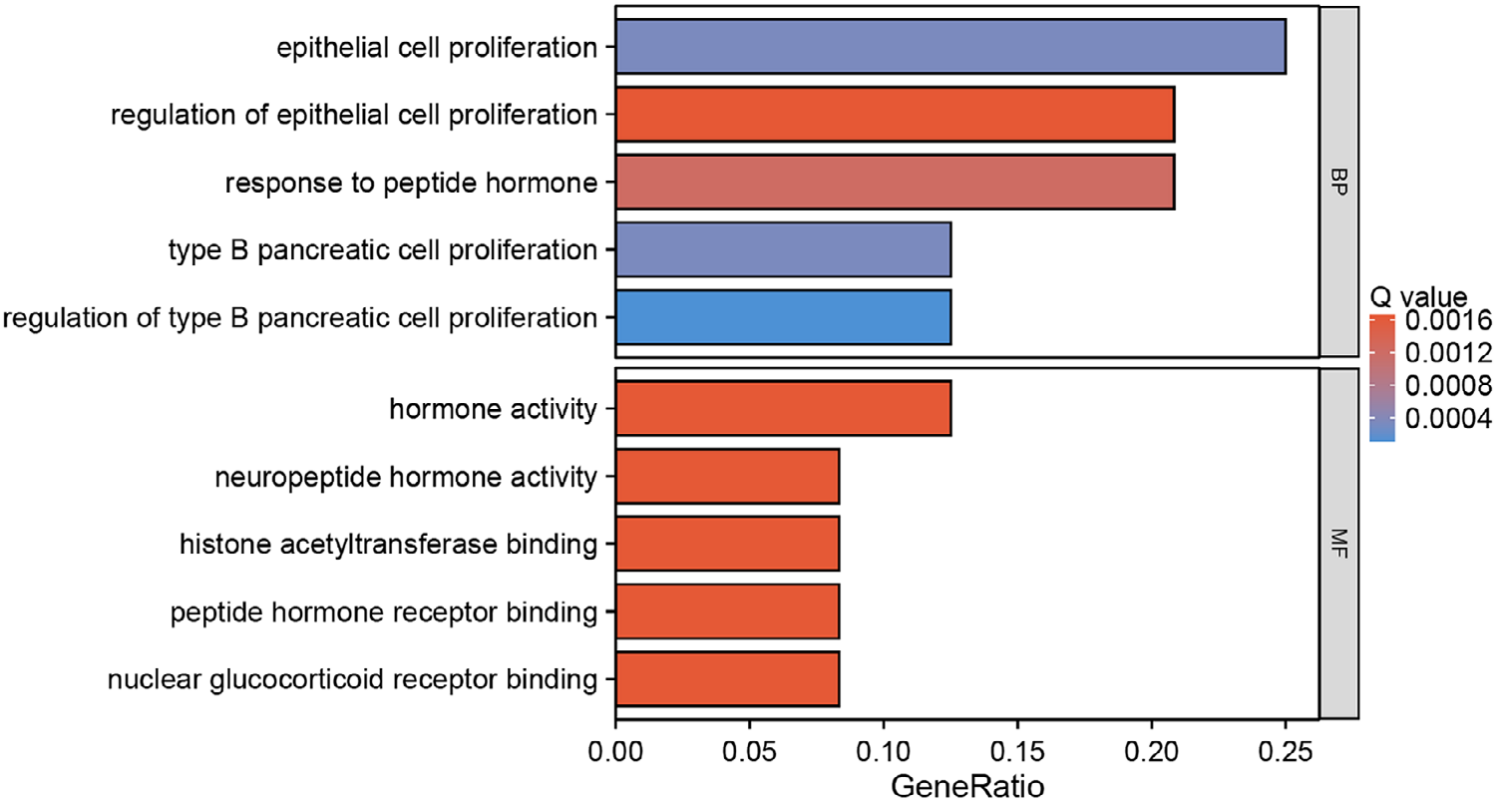
GO enrichment analysis of genes related to neuronal regeneration after IS. GO, gene ontology; IS, ischemic stroke.

To elucidate the GO/KEGG pathway in which the hub_gene may be implicated, the hub_gene was subjected to GO and KEGG enrichment analysis using the clusterProfiler package. On GO analysis results indicated that these genes were mainly enriched in “epithelial cell proliferation”, “regulation of epithelial cellproliferation”, “hormone activity”, “neuropeptide hormone activity”, and “neuropeptide hormone activity” in biological process (BP) and molecular function (MF) categories (Figure 3, Table 1). The KEGG pathway was not enriched in order to obtain results. The results indicate that hub_gene may play a role in promoting neuronal regeneration by regulating processes such as cell proliferation through the actions of hormones and neuropeptides, among other mechanisms.

**TABLE 1:** GO enrichment analysis of genes related to neuronal regeneration after IS.

Ontology	ID	Description	*q* value	geneID
BP	GO:0061469	Regulation of type B pancreatic cell proliferation	0.00010967	Nr4a3/Nr4a1/Sgpp2
BP	GO:0044342	Type B pancreatic cell proliferation	0.00032803	Nr4a3/Nr4a1/Sgpp2
BP	GO:0050673	Epithelial cell proliferation	0.00032803	Scg2/Dusp10/Nr4a3/Nr4a1/Irf6/Sgpp2
BP	GO:0043434	Response to peptide hormone	0.00122021	Hdac9/Egr1/Nr4a3/Nr4a1/Vgf
BP	GO:0050678	Regulation of epithelial cell proliferation	0.0016605	Scg2/Dusp10/Nr4a3/Nr4a1/Sgpp2
MF	GO:0035259	Nuclear glucocorticoid receptor binding	0.00164891	Nr4a3/Nr4a1
MF	GO:0051428	Peptide hormone receptor binding	0.00164891	Crh/Adcyap1
MF	GO:0005179	Hormone activity	0.00164891	Crh/Adcyap1/Vgf
MF	GO:0035035	Histone acetyltransferase binding	0.00164891	Egr1/Nr4a3
MF	GO:0005184	Neuropeptide hormone activity	0.00164891	Adcyap1/Vgf

### 3.3 PPI Analysis of Neuronal Regeneration After IS‐Related DEGs

**FIGURE 4:**
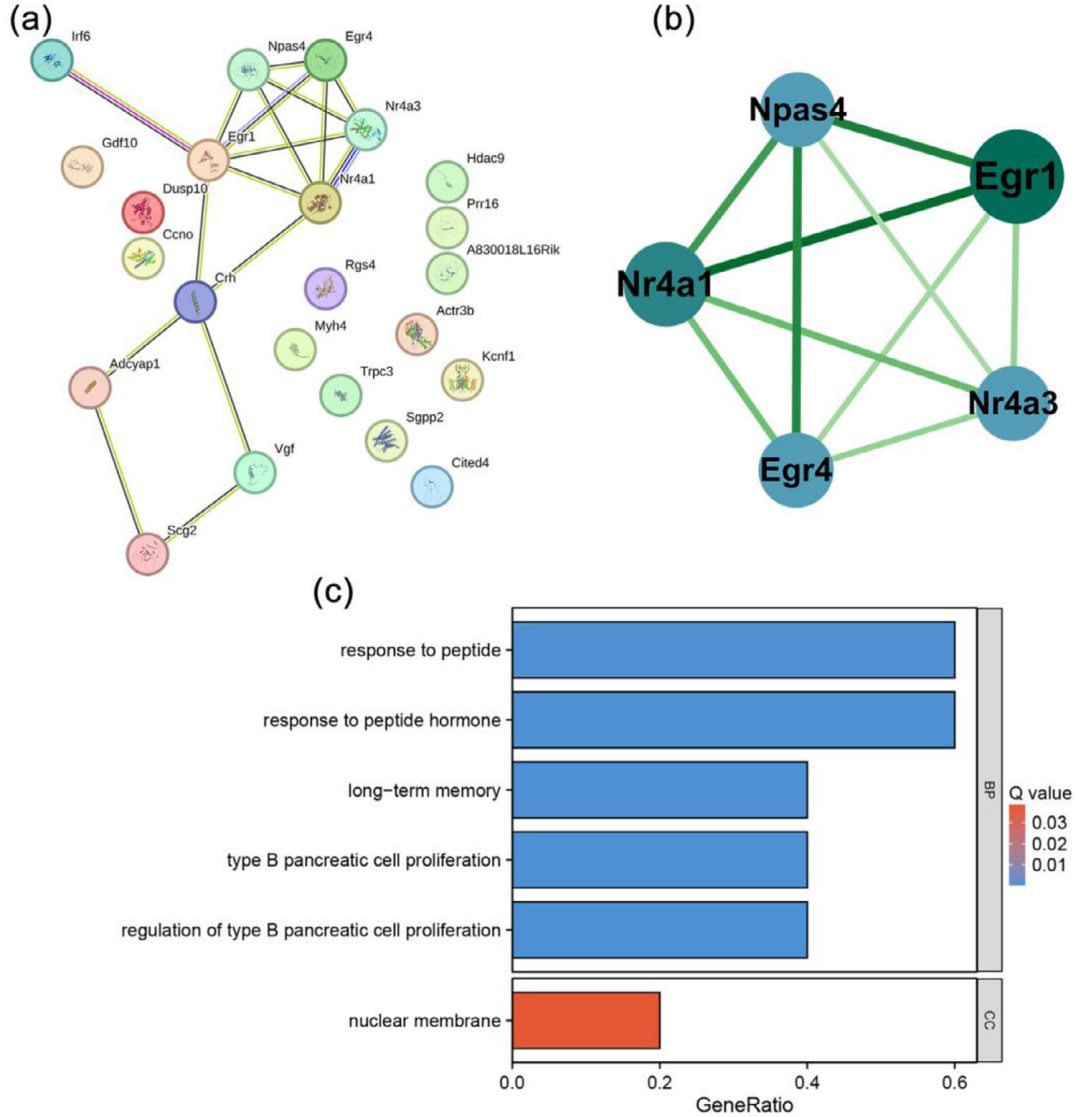
PPI network analysis of genes related to neuronal regeneration after IS. (a) PPI network of hub genes. (b) PPI network of key genes. (c) GO enrichment analysis results of key genes. GO, gene ontology; IS, ischemic stroke.

In order to identify the key genes associated with the hub_gene, we constructed a PPI network for the hub_gene using the STRING database and subsequently analyzed the PPI network using the Cytoscape software. The network comprised 10 nodes and 17 edges (Figure 4a), and the key cluster analysis was conducted using MCODE to identify key cluster 1, with a score of 5.0. This cluster included five nodes (Npas4, Nr4a3, Nr4a1, Egr4, and Egr1) and 10 edges (Figure 4b), representing the key genes. CytoNCA was used to calculate the PPI network parameters of the key genes, in which the genes with degree > 5 included Egr1 and Nr4a1. The results of the GO/KEGG enrichment analysis indicated that the principal genes were predominantly enriched in “response to peptide”, “response to peptide hormone”, “nuclear membrane” in BP and cellular components (CC) categories (Figure 4c). The results indicate that Egr1 and Nr4a1 may be the core genes influencing neuronal regeneration after IS, with hormone response representing a crucial pathway in this process.

### 3.4 Validation of Cellular Expression of Key Genes

**FIGURE 5:**
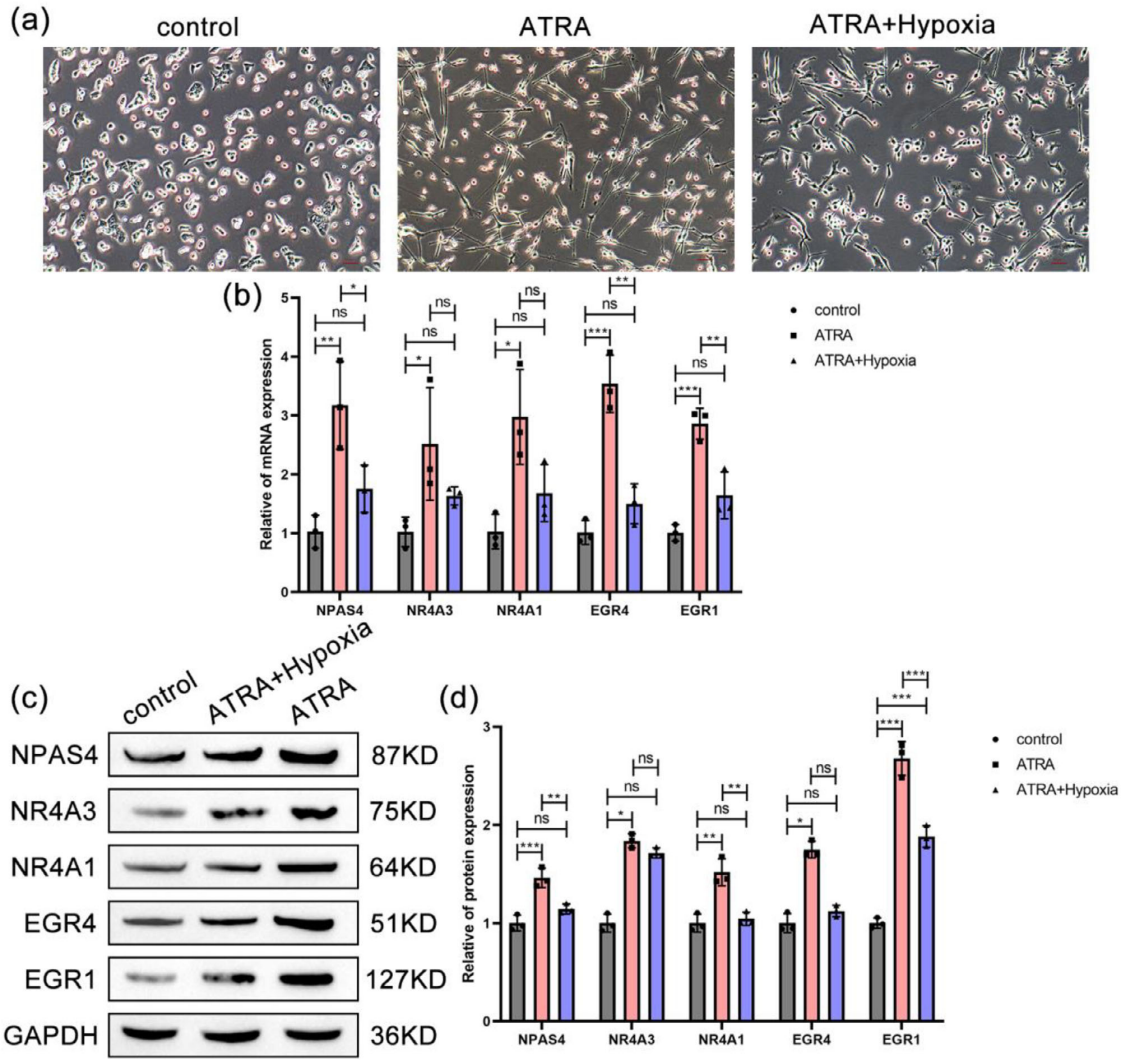
Verification of key gene expression in cells. (a) Neuronal‐like differentiation of SH‐SY5Y cells after induction. (b) Real‐time quantitative polymerase chain reaction (RT‐qPCR) detection of mRNA expression of key genes in SH‐SY5Y cells (^*^ *p* < 0.05; ^**^ *p* < 0.01; ^**^ *p* < 0.001). (c) Western Blot detection of protein expression of key genes in SH‐SY5Y cells. (d) Quantitative results of Western Blot analysis (^*^ *p* < 0.05; ^**^ *p* < 0.01; **p < 0.001).

Subsequently, ATAR was employed to induce hypoxia treatment in human SH‐SY5Y cells, thereby simulating the pathological environment of IS. This was undertaken to observe the differentiation of the cells into a neuronal‐like phenotype and to detect mRNA and protein levels, with a view to assessing the changes in key genes associated with neuronal induction. The results demonstrated that ATRA induces and promotes neuronal‐like differentiation of SH‐SY5Y cells. Under hypoxic treatment conditions, some SH‐SY5Y cells underwent neuronal‐like differentiation, but the overall level was inhibited (Figure 5a). The results from RT‐qPCR analysis demonstrated that compared to the control group, ATRA induced an upregulation of key gene mRNA expression in SH‐SY5Y cells, with the most significant increases observed for EGR4 and EGR1 (*p* < 0.001). Following hypoxic treatment, there were no significant differences in the expression levels of these key genes when compared to the control group. Moreover, hypoxic treatment significantly inhibited the ATRA‐induced expression levels of NPAS4 (*p* < 0.05), EGR4, and EGR1 (*p* < 0.01) (Figure 5b). The findings from the Western Blot analysis were consistent with those from RT‐qPCR. Compared to the control group, ATRA induced a significant upregulation of protein levels of key genes, with the most remarkable increase observed for EGR1 and NPAS4 proteins (*p* < 0.001). Hypoxic treatment impeded the upregulation of these key genes; however, the expression level of EGR1 was still significantly increased compared to the control group (*p* < 0.001). Additionally, hypoxic treatment significantly inhibited the upregulation of NPAS4 and NR4A1 (*p* < 0.01) compared to ATRA induction (Figure 5c,d). These results suggest that key genes are upregulated during the process of neuronal regeneration, but their expression levels are suppressed under hypoxic conditions, thereby hindering the progression of neuronal regeneration. Furthermore, the significant elevation of EGR1 protein levels even under hypoxic conditions indicates that EGR1 might play a more critical role in neuronal regeneration.

### 3.5 Analysis of Key Gene Transcription Factors and Interaction Networks

**FIGURE 6:**
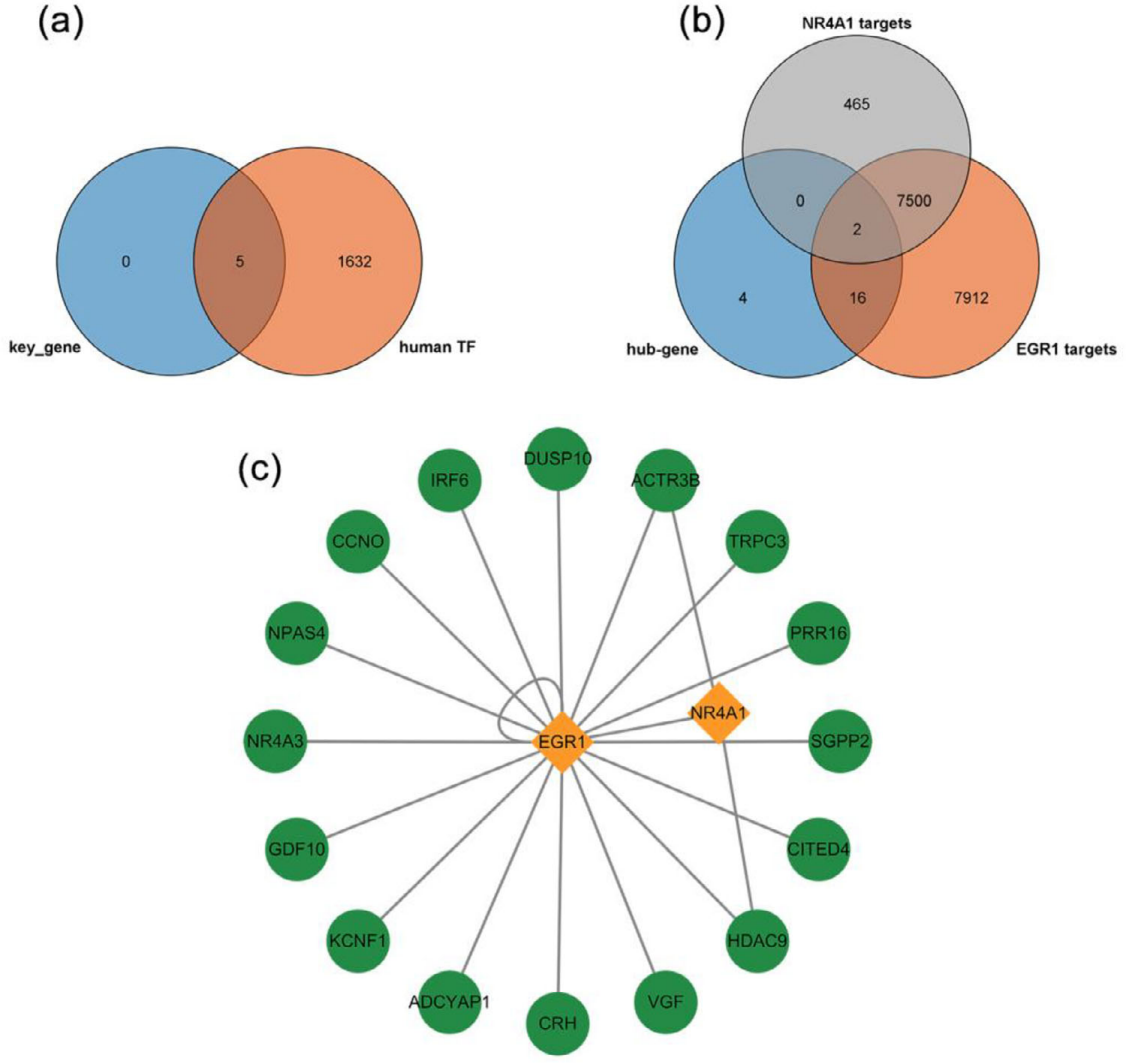
Analysis of key genes' transcription factors and interaction networks. (a) Venn diagram of key genes and human transcription factors. (b) Venn diagram of target genes of EGR1 and NR4A1 and hub genes. (c) Regulatory network of EGR1 and NR4A1.

Transcription factors play a pivotal role in regulating cellular gene expression. To elucidate the presence of transcription factors in the key genes, we obtained a list of human transcription factors from the AnimalTFDB v4.0 database. Subsequently, we plotted a Venn diagram (Figure 6a) to demonstrate that the key genes were all transcription factors. Subsequently, the target genes of the key genes were predicted by the TFLink database, and a Venn diagram was constructed to identify the target genes in hub_gene that may be regulated by the key genes. Among the key genes, NPAS4 did not predict the target genes, NR4A3 and EGR4 were not screened for possible regulation of hub_gene, and the NR4A1 screen yielded two potentially regulated hub_genea1, and the EGR1 screening yielded 18 potentially regulated hub_gene (Figure 6b). The visualization of the NR4A1/EGR1‐target gene regulatory network using Cytoscape revealed that EGR1, as a transcription factor, could target itself and target NR4A1. Additionally, EGR1 and NR4A1 were observed to co‐target genes, including ACTR3B and HDAC9 (Figure 6c). The results indicated that EGR1 and NR4A1 might be the core genes in the gene regulatory network related to neuronal regeneration after IS. Additionally, the results suggested that EGR1 might play the most critical regulatory roles as a transcription factor, which is in line with the previous analyses and detection results.

### 3.6 Overexpression of EGR1 Promotes Neuronal‐Like Differentiation of SH‐SY5Y Cells

**FIGURE 7:**
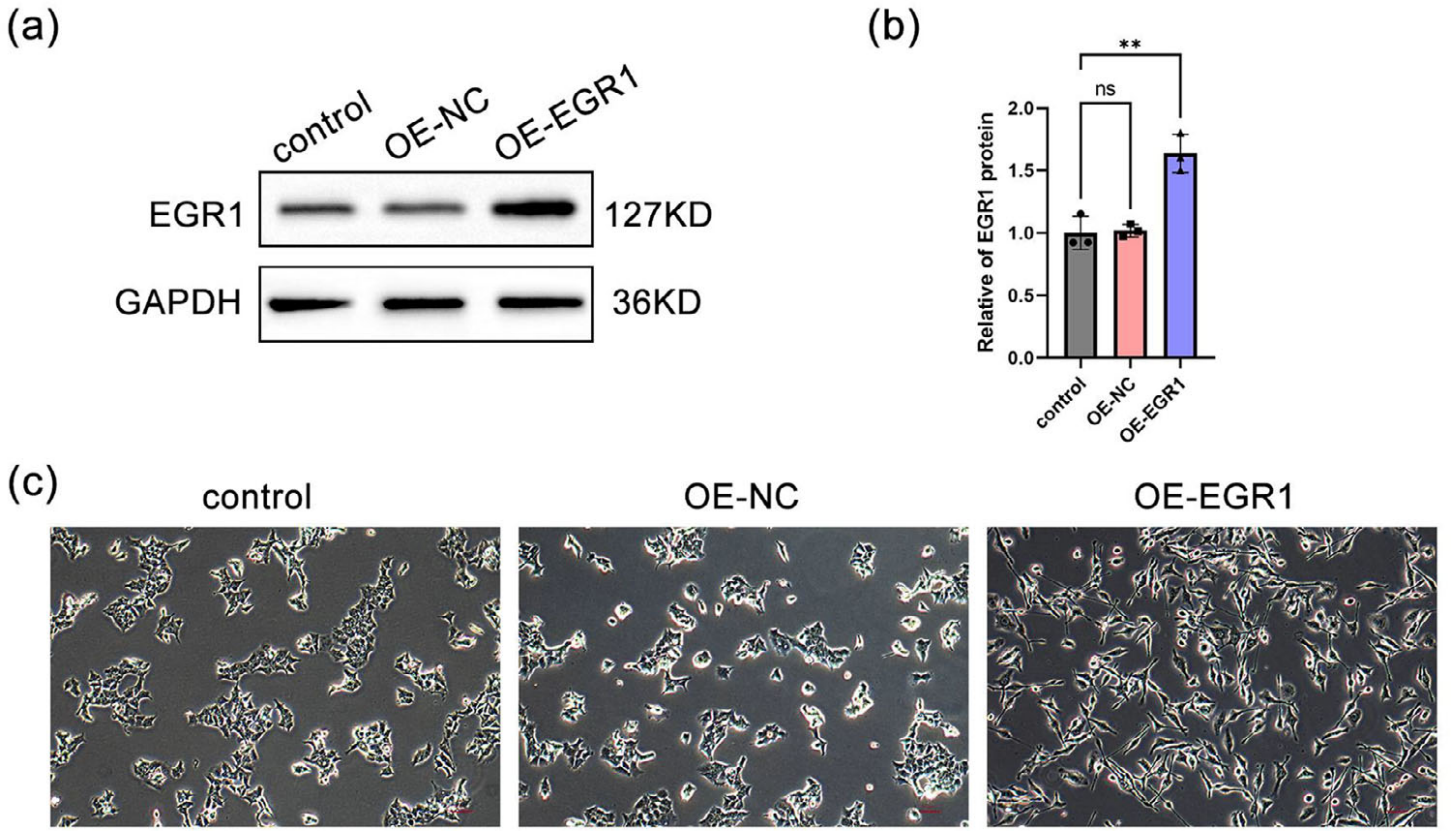
Overexpression of EGR1 promotes neuronal‐like differentiation of SH‐SY5Y cells. (a) Western Blot analysis of EGR1 expression levels in SH‐SY5Y cells. (b) The status of neuronal‐like differentiation in SH‐SY5Y cells.

We transfected SH‐SY5Y cells with the EGR1 overexpression vector, and the results of Western Blot analysis revealed that the OE‐EGR1 vector upregulated the expression level of EGR1 in SH‐SY5Y cells (Figure 7a). Subsequently, we observed the level of neuronal‐like differentiation in SH‐SY5Y cells following EGR1 overexpression. The results showed that after routine culture for 5 days, neither the control group nor the OE‐NC group exhibited apparent neuronal‐like differentiation. In contrast, SH‐SY5Y cells with overexpressed EGR1 displayed a certain degree of neuronal‐like differentiation, although the extent and characteristics of differentiation were relatively lower compared to ATRA induction (Figures 5a and 7b). These findings suggest that upregulation of EGR1 can promote neuronal‐like differentiation of SH‐SY5Y cells, albeit with limited effectiveness.

## 4 Discussion

IS is a complex disease characterized by the specificity of its etiological factors and the location of lesions. Targeted therapeutic strategies have always been a focus of researchers' attention. With the deepening of understanding of the disease, apart from early rapid‐response clinical treatment methods, management and treatment after IS have also begun to attract attention. The neurological damage caused by IS is an important reason for death and disability. Recent clinical and mechanistic studies on IS have revealed that the main mechanisms underlying neurological damage include: direct injury and death of neurons induced by ischemia and infarction (Chen et al. 2020); accumulation of reactive oxygen species (ROS) in neurons caused by ischemia, leading to oxidative stress that exacerbates neuronal damage and functional impairment (Hu et al. 2019); neuronal damage caused by inflammatory reactions induced by ischemia (Ren et al. 2020). These factors contribute to the loss of neurons in the affected area, making neuronal regeneration in the corresponding region particularly important. Previous studies have found the potential of inducing astrocytes, neural stem cells, to promote neuronal regeneration (Yin et al. 2019; Wang et al. 2024), but they still face challenges such as low induction success rates and survival rates. Therefore, identifying potential therapeutic targets for neuronal regeneration is of great significance.

Our study identified 24 differentially expressed hub genes associated with neuronal regeneration after IS. These genes were significantly suppressed after the occurrence of IS but were significantly upregulated in the regenerated neurons after induction, indicating their potential involvement in neuronal regeneration. It has been reported that brain ischemia can stimulate cell proliferation and neurogenesis in adult rodents and monkeys, and ischemia‐induced neurogenesis might persist for several months (Arvidsson et al. 2002; Kernie and Parent 2010). However, this endogenous neurogenesis is insufficient to repair the neurological damage caused by IS, which may be related to the inhibition of gene expression by ischemia, among others. Through GO enrichment analysis, we noticed that these genes are mainly enriched in processes such as epithelial cell proliferation regulation and hormone response, suggesting that these hub genes may participate in neuronal regeneration by responding to neuropeptide‐like hormones. Recent studies have found that neuropeptide‐like hormones and growth hormone‐releasing hormones have the potential to treat neurological injuries (Stoyanova and Lutz 2021), such as Liu report on a growth hormone‐releasing hormone agonist analog (MR‐409) that enhances the endogenous neurogenesis of mice subjected to brain ischemia (Liu et al. 2021). Additionally, other hormones in the body may also play a certain neuroprotective role, such as insulin (Chau et al. 2022). Our enrichment results are consistent with these research perspectives.

Our further analysis results indicate that NPAS4, NR4A3, NR4A1, EGR4, and EGR1 act as key genes, especially EGR1 and NR4A1 may serve as core genes in neuronal regeneration after IS. The protein encoded by EGR1 belongs to the C2H2‐type zinc finger protein family of the EGR family, acting as a transcriptional regulatory factor. Previous studies have proposed the role of EGR1 in neuronal development. Sofia found that Fingolimod could enhance the growth of primary neuronal neurites by upregulating EGR1 expression and changing the morphology of growth cones (Anastasiadou and Knöll 2016). Wang showed that EGR1, regulated by CCDC25, participates in the proliferation differentiation of neural stem cells and the maturation of neurons (Wang et al. 2023). Alex identified EGR1 as a candidate target of Notch signaling in human radial glial cells based on sequencing results (Pollen et al. 2014). Our analysis results further support the importance of EGR1. NR4A1 encodes a member of the steroid‐thyroid hormone‐retinoid receptor superfamily. Research has shown that the inhibition of NR4A1 expression mediates neuron injury in the oxygen‐glucose deprivation model (Liu et al. 2020), and progressive reduction of NR4A1 expression is associated with age‐dependent cognitive decline (Chen et al. 2024). Our analysis results also support the neurofunctional protective role of NR4A1. However, there are also studies proposing that NR4A1 promotes neuronal plasticity, but sustained expression or activity may be harmful (Jeanneteau et al. 2018), so further exploration of the relevant mechanisms of NR4A1 is still needed. Moreover, we observed changes in the expression of key genes and their relationship with neuronal‐like differentiation in cell models. We noticed that in SH‐SY5Y cells undergoing neuronal‐like differentiation, the expression levels of key genes were upregulated, suggesting that these key genes may be involved in the neurogenesis process. Under hypoxic conditions, however, the neuronal‐like differentiation of SH‐SY5Y cells was suppressed, which might be related to the inhibition of key gene expression levels. These validation data support our analysis results, indicating the potential of hub genes as therapeutic targets for neuronal regeneration after IS. Salvaging the expression levels or activation status of key genes may represent a means to promote neurogenesis after IS. Furthermore, we observed that compared to the control group, ATRA‐induced upregulation of EGR1 expression at both gene and protein levels. Although hypoxic treatment reduced the levels of EGR1, its expression at the protein level remained significantly higher than the control group (Figure 5d), while there was no significant difference in gene expression levels (Figure 5b). This discrepancy may be associated with post‐transcriptional modifications of EGR1, post‐translational modifications, mRNA, and protein degradation, as well as the duration of hypoxic treatment, among other possible factors. The underlying mechanisms could be critical in applying EGR1 to neuronal regeneration. Further analysis of the transcriptional regulatory network also suggests the potential of EGR1 and NR4A1, especially EGR1 as core targets.

Our study also has some limitations. First, the activation of endogenous neuroregeneration may not be sufficient to achieve the purpose of restoring neurological function. Research on neuroregeneration therapy related to clinical experiments also focuses more on exploring the potential of neural stem cell transplantation (Wang et al. 2024), although our study confirmed the potential of EGR1 and NR4A1 as therapeutic targets, we have not yet verified their roles in neural stem cells, so we cannot clearly identify the potential of core genes in the application field of neural stem cells. Secondly, the regulatory mechanisms of core genes have not been fully explored, so we cannot determine the feasible schemes and specific mechanisms for activating the expression and activity of EGR1 and NR4A1. Of course, this will also be the direction we will focus on in our future research.

In summary, in this study, we identified 24 hub genes related to neuronal regeneration after IS and verified the expression levels of five key genes. We hypothesize that EGR1 and NR4A1, as core genes, may regulate the regeneration of neurons after IS by influencing the response of hub genes to hormones such as neuropeptides.

## 5 Conclusion

The EGR1 may represent promising avenues for therapeutic intervention in the context of neuronal regeneration after IS.

## Author Contributions

**Xiao‐Li Min**: conceptualization, methodology, data curation, writing–original draft, writing–review and editing. **Li Guo**: data curation, conceptualization, writing–original draft, writing–review and editing. **Zhenyu Wang**: investigation, data curation. **Lei Zhao**: software, validation. **Chenglong Shi**: project administration, software. **Xiaoyong Liu**: investigation, formal analysis. **Zhen Wang**: investigation, visualization. **Fei‐Fei Shang**: funding acquisition, resources, supervision, writing–review and editing. **Jiaping Wang**: supervision, funding acquisition, writing–review and editing, resources.

### Peer Review

The peer review history for this article is available at https://publons.com/publon/10.1002/brb3.70377.

## Data Availability

The data that support the findings of this study are available from the corresponding author upon reasonable request.

## References

[brb370377-bib-0001] Anastasiadou, S. , and B. Knöll . 2016. “The Multiple Sclerosis Drug Fingolimod (FTY720) Stimulates Neuronal Gene Expression, Axonal Growth and Regeneration.” Experimental Neurology 279: 243–260. 10.1016/j.expneurol.2016.03.012. 26980486

[brb370377-bib-0002] Anfray, A. , C. Brodin , A. Drieu , et al. 2021. “Single‐ and Two‐Chain Tissue Type Plasminogen Activator Treatments Differentially Influence Cerebral Recovery After Stroke.” Experimental Neurology 338: 113606. 10.1016/j.expneurol.2021.113606. 33453214

[brb370377-bib-0003] Arvidsson, A. , T. Collin , D. Kirik , Z. Kokaia , and O Lindvall . 2002. “Neuronal Replacement From Endogenous Precursors in the Adult Brain After Stroke.” Nature Medicine 8, no. 9: 963–970. 10.1038/nm747. 12161747

[brb370377-bib-0004] Bailey, E. L. , C. Smith , C. L. Sudlow , and J. M Wardlaw . 2012. “Pathology of Lacunar Ischemic Stroke in Humans–A Systematic Review.” Brain Pathology 22, no. 5: 583–591. 10.1111/j.1750-3639.2012.00575.x. 22329603 PMC8057646

[brb370377-bib-0005] Barker, R. A. , M. Götz , and M Parmar . 2018. “New Approaches for Brain Repair‐From Rescue to Reprogramming.” Nature 557, no. 7705: 329–334. 10.1038/s41586-018-0087-1. 29769670

[brb370377-bib-0006] Chau, D. D. , W. Li , W. W. R. Chan , et al. 2022. “Insulin Stimulates Atypical Protein Kinase C‐Mediated Phosphorylation of the Neuronal Adaptor FE65 to Potentiate Neurite Outgrowth by Activating ARF6‐Rac1 Signaling.” FASEB Journal 36, no. 11: e22594. 10.1096/fj.202200757R. 36250347

[brb370377-bib-0007] Chen, C. , Y. Wu , J. Li , et al. 2023. “TBtools‐II: A “One for All, All for One” Bioinformatics Platform for Biological Big‐Data Mining.” Molecular Plant 16, no. 11: 1733–1742. 10.1016/j.molp.2023.09.010. 37740491

[brb370377-bib-0008] Chen, J. , Z. Zhang , Y. Liu , et al. 2024. “Progressive Reduction of Nuclear Receptor Nr4a1 Mediates Age‐Dependent Cognitive Decline.” Alzheimer's and Dementia 20, no. 5: 3504–3524. 10.1002/alz.13819. PMC11095431 38605605

[brb370377-bib-0009] Chen, Y. C. , N. X. Ma , Z. F. Pei , et al. 2020. “A NeuroD1 AAV‐Based Gene Therapy for Functional Brain Repair After Ischemic Injury Through In Vivo Astrocyte‐to‐Neuron Conversion.” Molecular Therapy 28, no. 1: 217–234. 10.1016/j.ymthe.2019.09.003. 31551137 PMC6952185

[brb370377-bib-0010] Encinas, M. , M. Iglesias , Y. Liu , et al. 2000. “Sequential Treatment of SH‐SY5Y Cells With Retinoic Acid and Brain‐Derived Neurotrophic Factor Gives Rise to Fully Differentiated, Neurotrophic Factor‐Dependent, Human Neuron‐Like Cells.” Journal of Neurochemistry 75, no. 3: 991–1003. 10.1046/j.1471-4159.2000.0750991.x. 10936180

[brb370377-bib-0011] Feigin, V. L. , M. H. Forouzanfar , R. Krishnamurthi , et al. 2014. “Global and Regional Burden of Stroke During 1990–2010: Findings From the Global Burden of Disease Study 2010.” Lancet 383, no. 9913: 245–254. 10.1016/s0140-6736(13)61953-4. 24449944 PMC4181600

[brb370377-bib-0012] Hankey, G. J. 2017. “Stroke.” Lancet 389, no. 10069: 641–654. 10.1016/s0140-6736(16)30962-x. 27637676

[brb370377-bib-0013] Hilkens, N. A. , B. Casolla , T. W. Leung , and F. E. de Leeuw . 2024. “Stroke.” Lancet 403, no. 10446: 2820–2836. 10.1016/s0140-6736(24)00642-1. 38759664

[brb370377-bib-0014] Hu, B. , J. Pei , and C. Wan , et al. 2024. “Mechanisms of Postischemic Stroke Angiogenesis: A Multifaceted Approach.” Journal of Inflammation Research 17: 4625–4646. 10.2147/jir.S461427. 39045531 PMC11264385

[brb370377-bib-0015] Hu, X. , D. Wu , X. He , et al. 2019. “circGSK3β Promotes Metastasis in Esophageal Squamous Cell Carcinoma by Augmenting β‐Catenin Signaling.” Molecular Cancer 18, no. 1: 160. 10.1186/s12943-019-1095-y. 31722716 PMC6854808

[brb370377-bib-0016] Jeanneteau, F. , C. Barrère , M. Vos , et al. 2018. “The Stress‐Induced Transcription Factor NR4A1 Adjusts Mitochondrial Function and Synapse Number in Prefrontal Cortex.” Journal of Neuroscience 38, no. 6: 1335–1350. 10.1523/jneurosci.2793-17.2017. 29295823 PMC5815341

[brb370377-bib-0017] Kernie, S. G. , and J. M Parent . 2010. “Forebrain Neurogenesis After Focal Ischemic and Traumatic Brain Injury.” Neurobiology of Disease 37, no. 2: 267–274. 10.1016/j.nbd.2009.11.002. 19909815 PMC2864918

[brb370377-bib-0018] Kleindorfer, D. O. , A. Towfighi , S. Chaturvedi , et al. 2021. “2021 Guideline for the Prevention of Stroke in Patients With Stroke and Transient Ischemic Attack: A Guideline From the American Heart Association/American Stroke Association.” Stroke; A Journal of Cerebral Circulation 52, no. 7: e364–e467. 10.1161/str.0000000000000375. 34024117

[brb370377-bib-0019] Levine, D. A. , A. T. Galecki , K. M. Langa , et al. 2015. “Trajectory of Cognitive Decline After Incident Stroke.” Jama 314, no. 1: 41–51. 10.1001/jama.2015.6968. 26151265 PMC4655087

[brb370377-bib-0020] Liu, L. L. , S. Qiao , M. L. Wang , et al. 2020. “MiR224‐5p Inhibitor Restrains Neuronal Apoptosis by Targeting NR4A1 in the Oxygen‐Glucose Deprivation (OGD) Model.” Frontiers in Neuroscience 14: 613. 10.3389/fnins.2020.00613. 32670010 PMC7330102

[brb370377-bib-0021] Liu, Y. , J. Yang , and X. Che , et al. 2021. “Agonistic Analog of Growth Hormone‐Releasing Hormone Promotes Neurofunctional Recovery and Neural Regeneration in Ischemic Stroke.” Proceedings of the National Academy of Sciences USA 118, no. 47. 10.1073/pnas.2109600118. PMC8617525 34782465

[brb370377-bib-0022] Ninomiya, I. , A. Koyama , Y. Otsu , O. Onodera , and M Kanazawa . 2023. “Regeneration of the Cerebral Cortex by Direct Chemical Reprogramming of Macrophages Into Neuronal Cells in Acute Ischemic Stroke.” Frontiers in Cellular Neuroscience 17: 1225504. 10.3389/fncel.2023.1225504. 37636590 PMC10457112

[brb370377-bib-0023] Pollen, A. A. , T. J. Nowakowski , J. Shuga , et al. 2014. “Low‐Coverage Single‐Cell mRNA Sequencing Reveals Cellular Heterogeneity and Activated Signaling Pathways in Developing Cerebral Cortex.” Nature Biotechnology 32, no. 10: 1053–1058. 10.1038/nbt.2967. PMC4191988 25086649

[brb370377-bib-0024] Powers, W. J. 2020. “Acute Ischemic Stroke.” New England Journal of Medicine 383, no. 3: 252–260. 10.1056/NEJMcp1917030. 32668115

[brb370377-bib-0025] Qin, C. , S. Yang , Y. H. Chu , et al. 2022. “Signaling Pathways Involved in Ischemic Stroke: Molecular Mechanisms and Therapeutic Interventions.” Signal Transduction and Targeted Therapy 7, no. 1: 215. 10.1038/s41392-022-01064-1. 35794095 PMC9259607

[brb370377-bib-0026] Ren, X. , H. Hu , I. Farooqi , and J. W Simpkins . 2020. “Blood Substitution Therapy Rescues the Brain of Mice From Ischemic Damage.” Nature Communications 11, no. 1: 4078. 10.1038/s41467-020-17930-x. PMC7447645 32843630

[brb370377-bib-0027] Stoyanova, I. , and D Lutz . 2021. “Ghrelin‐Mediated Regeneration and Plasticity After Nervous System Injury.” Frontiers in Cell and Developmental Biology 9: 595914. 10.3389/fcell.2021.595914. 33869167 PMC8046019

[brb370377-bib-0028] Wang, C. , J. Qin , J. Jiao , and F Ji . 2023. “Ccdc25 Regulates Neurogenesis During the Brain Development.” Developmental Neurobiology 83, no. 3‐4: 91–103. 10.1002/dneu.22911. 37092777

[brb370377-bib-0029] Wang, S. , Q. He , Y. Qu , et al. 2024. “Emerging Strategies for Nerve Repair and Regeneration in Ischemic Stroke: Neural Stem Cell Therapy.” Neural Regeneration Research 19, no. 11: 2430–2443. 10.4103/1673-5374.391313. 38526280 PMC11090435

[brb370377-bib-0030] Yin, J. C. , L. Zhang , N. X. Ma , et al. 2019. “Chemical Conversion of Human Fetal Astrocytes Into Neurons Through Modulation of Multiple Signaling Pathways.” Stem Cell Reports 12, no. 3: 488–501. 10.1016/j.stemcr.2019.01.003. 30745031 PMC6409415

